# The epichaperome: the power of many as the power of one

**DOI:** 10.18632/oncoscience.321

**Published:** 2016-10-26

**Authors:** Wang Tai, Monica L. Guzman, Gabriela Chiosis

**Affiliations:** Program in Chemical Biology, Sloan Kettering Institute, New York, New York 10065, USA; Breast Cancer Service, Department of Medicine, Memorial Sloan Kettering Cancer Center, New York, New York 10065, USA

**Keywords:** chaperome networks, HSP90, chronic stress, PU-H71, epichaperome

In a new study in cancer, we now recognize a major driver of the disease. This discovery is intriguing in that this cancer driver crosses several boundaries; it is found in half of the total cancer patient population, and it spans all cancers regardless of tissue of origin or cancer gene mutation. This cancer driver is a novel chaperome entity, present in the disease state via the transformation and banding together of individual chaperome members found in the healthy state [1, 2].

The chaperome is a family of individuals. There are the chaperones, such as the heat shock proteins (HSPs) – the HSP90s, the HSP70s, the HSP60 and HSP110s, and the small HSPs. Let us not forget the co-chaperones, which help chaperones, but which sometimes are chaperones in their own right. There are many other helpers as well, such as the isomerases and scaffolding proteins. Some chaperomes fold, some transport, some stabilize a protein or proteins we refer to as ‘client’ or ‘clients’ [3].

In a new study in cancer we have come to see the chaperome as individuals who have lost their personality to become one. We have come to understand the chaperome as a unique entity rather than a family of many [1].

**Figure 1:**
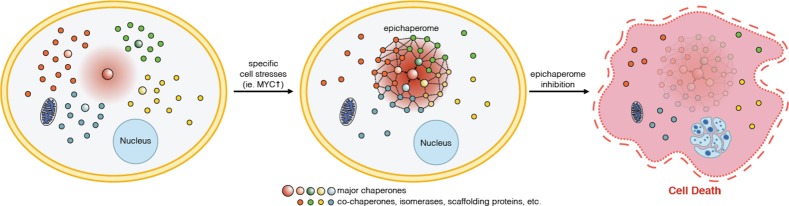
Schematic showing the re-wiring of the chaperome into the epichaperome network, following a specific cell stress. Inhibition of one of the epichaperome components dismantles the network. When the network is key to maintain viability in such cancer cell, epichaperome dismantling results in cell death.

This entity we coined as the epichaperome (Figure 1). In cancer cells, the chaperome goes rogue and delivers proliferative and survival advantages so cells can grow into the tumors we dread. For decades, researchers thought that fighting back with drugs targeting chaperones would be a sure bet for cancer. But in spite of much effort from pharma to turn chaperone inhibitors into cancer drugs, the road to success has been slow and littered with failures [4].

Why don't all patients respond to cancer drugs that bring a chaperone function to a halt? To provide an explanation to this paradox we probed two subsets of cancer cells representing the two types of cancer patients: those who respond and those who don't. The first set comprised cancer cells that died when the activity of the most abundant chaperone in the human cell, HSP90, was inhibited using an HSP90-inhibitor drug. The second subset included cancer cells that survived.

To address this question we also employed a variety of novel tools and methods; these maintained the endogenous native state of tumors and thus queried the chaperome in its natural state [5-8].

What we found is that in the first set, where tumor cells were effectively killed by inhibition of HSP90, the chaperome had become “communal”, and individual chaperome members banded together and behaved as one entity. This was in response to being overwhelmed by high demand for functions in response to drastic changes in the cellular milieu, whereby HSP90 reached over to other chaperome members and formed highly interconnected networks. Together, they shared the burden of work.

Perhaps the most intriguing revelation of this study, as it gives us an upper-hand in the war against cancer, was that the efficient “communal” team that gave the first subset of cancer cells superior growth capacity in turn also made them vulnerable to HSP90-inhibitors. Specifically, when one piece of the highly connected communal chaperome network was knocked out by an inhibitor drug, the entire network fell apart, just like a string of dominos, and cancer cell death ensued.

In the second set of cancer cells where tumors could not be inhibited by an HSP90-inhibitor drug, the chaperome remained “solitary”, i.e. behaved as individual chaperome and/or chaperome machinery; the individual chaperomes needed not band together to perform cellular functions in these cancer cells. Hence, the chaperome as a family did not dismantle and the cell could not be killed by taking out “solitary” chaperome members with inhibitor drugs.

What made the chaperome in certain cancer cells to become “communal” and therefore susceptible to inhibitor drugs, while in other cancer cells its individuals remained “solitary” and could not be knocked out to cause cell death? This was a question that we tackled by looking for differences between responders and non-responders to inhibitor drugs. Our work revealed that in all cancers out there, half were of the type that contained the “communal” chaperome teams and the other half contained “solitary” chaperomes, but that there was no association of either group with known cancer genetic mutations, sites where cancer originated, or cancer type.

What was a reason for the difference, then? The answer came when we looked for what could create the network of chaperomes. It was MYC, a cancer-causing gene behind the most aggressive types of cancer. If MYC was artificially added into a cancer cell that could not be killed with HSP90 inhibitors, we saw HSP90 go “communal” with the other chaperomes, and the cancer cell then became susceptible to cell death by HSP90 inhibitors. Conversely, if MYC was removed, HSP90 went back to being “solitary” and the cacner cell became immune to HSP90 inhibition.

This work helps us to better understand how to distinguish and predict patients who will respond verses those who will not respond to HSP90 inhibitor drugs, and to improve our approach to designing new drugs. It also gives us an understanding on how best to combine HSP90 inhibitors with other drugs, for cancer treatment.

“Communal” behavior to benefit survival is reflected in nature at the organismal level. Ants and bees work together in colonies, and their cooperative behavior determines the survival of the entire group. The colony becomes and functions differently from an individual ant or an individual bee. Their network becomes analogous to a single organism, just like the individual chaperomes forming communal entities in aggressive cancers become one.

Humans also use such mechanism; we build buildings from individual bricks – the building becomes and entity, both functionally and structurally, that is distinct from the many individual bricks. Same, we as people, build a society that is formed of many individuals; the society, while caries the traits of each individual, it does manifest as an entity that is distinct from each of us. Nature apparently uses similar patterns at different levels in the organization of life – what can be found in a cell is also repeated in multicellular organisms and multiorganism communities. It is therefore safe to assume that re-wiring of the chaperome is not limited to the stress imposed by MYC. It is possible that other chronic stresses imposed by changes in cellular milieu, whether manifested and driven by changes in the proteome or the cellular microenvironment, may lead to epichaperome formation. Will the epichaperome always manifest by the rewiring of the same chaperome components? Unlikely. The building blocks of the epichaperome and the structures it forms will likely conform and evolve based on cellular demands and new obstacles that it needs to overcome - we build a fortress to defend against an enemy and a house for people to live in.
